# Relationship between iron overload caused by abnormal hepcidin expression and liver disease: A review

**DOI:** 10.1097/MD.0000000000033225

**Published:** 2023-03-17

**Authors:** Haoran Zheng, Fan Yang, Kaige Deng, Jiaxin Wei, Zhenting Liu, Yong-Chang Zheng, Haifeng Xu

**Affiliations:** a Chinese Academy of Medical Sciences & Peking Union Medical College, Beijing, People’s Republic of China; b Division of Liver Surgery, Department of Surgery, Peking Union Medical College Hospital, Peking Union Medical College & Chinese Academy of Medical Sciences, Beijing, China; c Department of Emergency, Peking Union Medical College Hospital, Chinese Academy of Medical Sciences & Peking Union Medical College, Beijing, China.

**Keywords:** hepcidin, homeostasis, iron overload, liver disease

## Abstract

Iron is essential to organisms, the liver plays a vital role in its storage. Under pathological conditions, iron uptake by the intestine or hepatocytes increases, allowing excess iron to accumulate in liver cells. When the expression of hepcidin is abnormal, iron homeostasis in humans cannot be regulated, and resulting in iron overload. Hepcidin also regulates the release of iron from siderophores, thereby regulating the concentration of iron in plasma. Important factors related to hepcidin and systemic iron homeostasis include plasma iron concentration, body iron storage, infection, inflammation, and erythropoietin. This review summarizes the mechanism and regulation of iron overload caused by hepcidin, as well as related liver diseases caused by iron overload and treatment.

## 1. Introduction

Iron is a key cofactor in many biochemical activities. Most mammalian cells obtain iron from plasma transferrin (Tf). Iron-containing Tf binds to transferrin receptor 1 (TfR1) on the cell surface and transports its contents into the cytoplasm by endocytosis. Human Tf is a 76-kDa glycoprotein that is mainly produced in the liver and has a half-life of approximately 8 days in serum.^[1]^ All vertebrates have functional Tf or ovotransferrin, which has iron-binding and antibacterial properties in reptiles and birds.^[2]^ When the tissue demand for iron increases, iron deficiency triggers the transcription of TfR1 by hypoxia-inducible factors. Moreover, the combination of iron-regulatory proteins and their iron-responsive elements achieves posttranscriptional stability of TfR1 mRNA.^[3,4]^

Approximately 1 mg of iron is excreted through feces, urine, sweat, skin daily, and most of this is absorbed through the duodenum. However, iron uptake by the intestine or hepatocytes increases under pathological conditions. Excess iron accumulates in hepatocytes, which exerts its toxicity by catalyzing the production of reactive oxygen species (ROS).^[5]^ In the Fenton reaction (Fe^2+^+H_2_O_2_→ Fe^3+^+ hydroxyl radicals [OH] + OH^-^), a large number of toxic OH are produced.^[6,7]^ OH induce lysosomal, cytoplasmic, nuclear, mitochondrial membrane damage, and activating caspases and associated cell apoptosis and excessive oxidation of aliphatic chains to cause cell damage.^[8]^

Iron overload is generally defined as an increase of more than 5 g of iron in the whole body.^[9]^ When too much iron is in the body, storage proteins denature, releasing large amounts of iron into the cytoplasm of liver cells. Therefore, the liver is most likely to be affected by iron overload. If iron overload exceeds the ability of hepatocytes to isolate iron safely, ferritin subunits denature, releasing ionic iron into the cytoplasm of hepatocytes. Hepcidin is a peptide hormone with 25 amino acids rich in cysteine. It is formed by a simple hairpin structure that is stabilized by 4 disulfide bonds and is the system’s main regulator of iron homeostasis. Hepcidin is released from hepatocytes and binds to membrane ferroportin (FPN), inducing the internalization, ubiquitination, and degradation of FPN and thus affecting cell iron release and serum iron concentration. FPN is highly expressed in cell types that play a key role in iron metabolism, including the basolateral membrane of duodenal enterocytes, liver and spleen macrophages, and placental syncytiotrophoblasts. Under iron overload, the body releases substances that affect FPN, such as hepcidin, to reduce iron outflow. Otherwise, as a major iron-regulatory hormone, hepcidin controls plasma iron levels by preventing intestinal iron absorption and macrophage iron circulation. The liver is the main source of hepcidin, which macrophages, islet cells, and adipose tissue can also synthesize. By controlling the number of FPN molecules on the cell surface, the iron outflow from the cells is regulated by a negative feedback mechanism.^[10]^ Many forms of iron overload are characterized by low levels of hepcidin, which forms the basis of this paper: the abnormal regulation of hepcidin expression in the context of liver disease is related to the imbalance of iron metabolism.

## 2. Production

Hepcidin is mainly produced by hepatocytes near the portal vein and Kupffer cells and, to a lesser extent, by macrophages and adipocytes.^[11]^ Since the amount of hepcidin expressed by hepatocytes is 15 to 1500 times higher than that of other cells, hepatocytes are the most crucial source of hepcidin.

First, liver cells indirectly respond to iron by producing iron-regulated bone morphogenetic protein (BMP) via hepatic sinusoidal endothelial cells.^[12]^ The BMP pathway is essential for the expression of hepcidin. BMPs represent a large subfamily of the transforming growth factor b ligand superfamily, which shares a common signal transduction model.^[13]^ The BMP subfamily regulates hepcidin expression by signaling through receptor-activated Smads. The source of BMP6 in the liver is liver sinusoidal endothelial cells,^[14]^ and iron load stimulates the production of BMP6. Then, BMP6 binds to BMP II receptors, phosphorylates BMP I receptors, activates the Smad1/5/8 pathway, forms isomer complexes with Smad4, translocate to the nucleus, and induces the transcription of hepcidin.^[15,16]^ Second, hepatocytes can also sense iron directly through the expression of TfR1, transferrin receptor 2 (TfR2), and HFE proteins on the cell surface. With the increase in serum iron concentration, the expression of TfR2 exceeds that of TfR1, and Tf-Fe (III) simultaneously combines with TfR1 and TfR2 to increase the stability of TfR2.^[17]^ Moreover, HFE regulates iron absorption mainly by affecting the expression of hepcidin, which is the primary way to regulate iron metabolism.^[18]^

In addition to the main pathway in hepatocytes – BMP-SMAD – other factors that control hepcidin expression include inflammation, anemia, and hypoxia. These factors can be powerful inducers and suppressors of hepcidin expression when they transcend the BMP-SMAD pathway.

**Figure 1.**
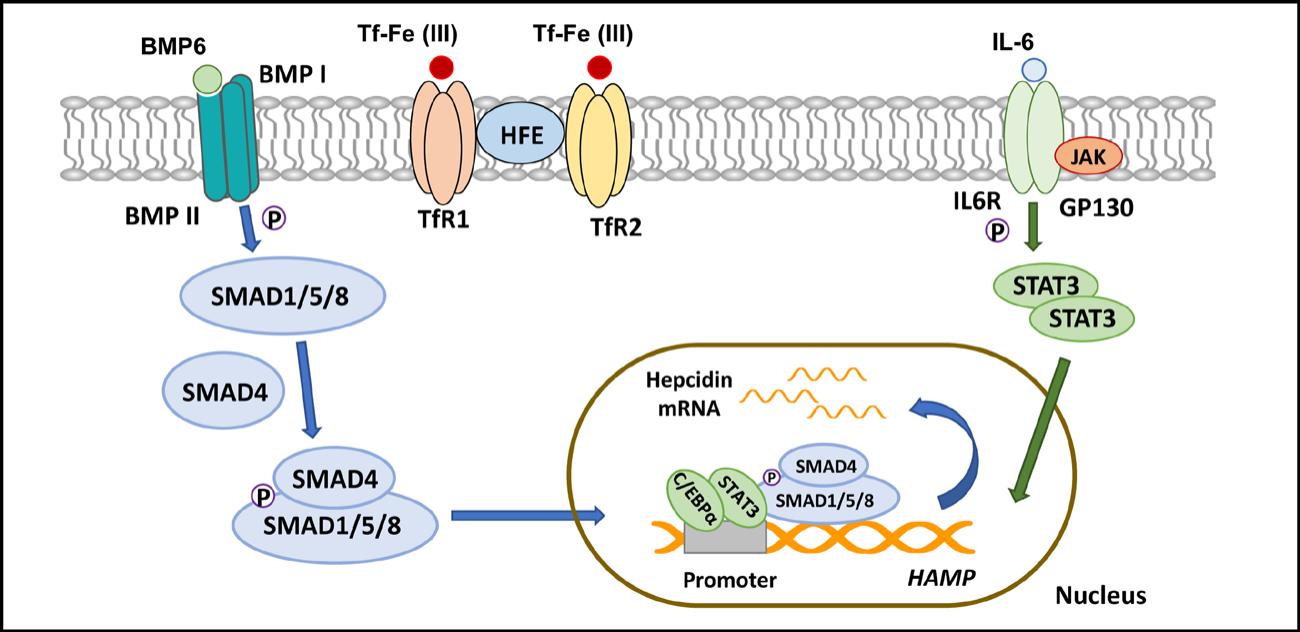
Multiple pathways regulate hepcidin expression. Upon stimulation of BMP6 production by iron loading, BMP6 binds to the BMP II receptor, phosphorylates the BMP I receptor, activates the Smad1/5/8 pathway, forms an isoform complex with Smad4, translocate to the nucleus, induces hepcidin transcription. Second, hepatocytes can also directly sense iron through the expression of TfR1, TfR2, and HFE proteins on the cell surface. In addition, the HAMP promoter contains binding sites for STAT-3 and C/EBPα, and these 2 pathways converge in the process of inflammation. BMP = bone morphogenetic protein, C/EBPα = CCAAT enhancer-binding protein α, STAT-3 = the signal transducer and activator of transcription 3, TfR1 = transferrin receptor 1, TfR2 = transferrin receptor 2.

The synthesis of hepcidin increases during infection or inflammation. During inflammation, the induction of hepcidin is usually mediated by interleukin-6 (IL-6). Elizabeta^[19]^ proved that IL-6 is necessary to induce hepcidin during inflammation, and the cytokine itself can rapidly induce iron deficiency anemia. Some crosstalk between the signal transducer and activator of the transcription 3 (STAT-3) signaling pathway induced by IL-6 and the BMP/SMAD signaling pathway has been confirmed. For example, the SMAD binding site on the hepcidin promoter is still critical for IL-6-mediated hepcidin expression,^[20]^ and hepcidin is less responsive to IL-6 in liver-specific gene Smad4 knockout mice.^[21]^ Another vital transcription factor regulating the expression of ferritin is CCAAT enhancer-binding protein α (C/EBPα). The HAMP promoter contains binding sites for STAT-3 and C/EBPα, and the 2 pathways converge in the process of inflammation. (Fig. 1).

Due to the high iron demand of hemoglobin synthesis, erythropoiesis dominates the regulation of iron metabolism to adapt to the higher iron demand of stress-induced red cell formation. Iron overload is usually associated with an enlarged size of red blood cells and higher cellular hemoglobin concentrations.^[22]^ This disease with simultaneous iron overload and increased erythropoiesis shows lower hepcidin expression than expected, despite increased iron storage. The expression of hepcidin is related to iron overload in these diseases and predicts the existence of “red-like factors” that regulate iron metabolism.

To explore the mechanism by which erythropoietin (EPO) regulates iron metabolism, it is necessary to separately consider EPO, hypoxia, anemia, reticulosis, and erythropoiesis. Previous experiments have shown that venotomy, administration of EPO, and hemolysis all lead to a decrease in the expression of hepcidin.

**Figure 2.**
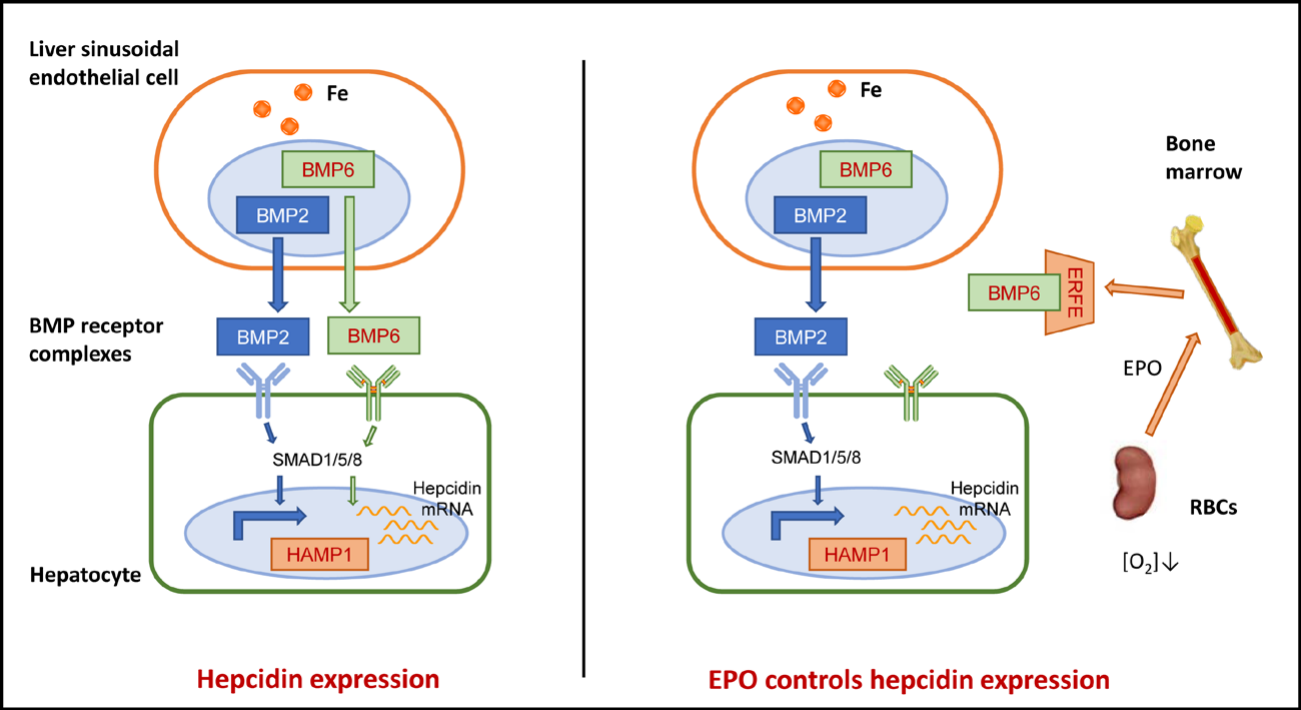
BMP/SMAD signaling modulates hepcidin expression: Activation of the transcriptional response, including hepcidin (HAMP), is mediated by BMP receptors on hepatocyte cell membranes that phosphorylate cytosolic SMADs (SMAD1, SMAD5, and SMAD8) that move into the nucleus complexed with SMAD4. ERFE inhibits BMP5, BMP6, and BMP7-induced induction of hepcidin expression. BMP = bone morphogenetic protein, ERFE = erythroferrone.

In the case of active red blood cell production, the production of hepcidin is inhibited. In addition, the concentration of serum erythroferrone (ERFE) in patients with blood loss, EPO loss, or β-thalassemia increases, and the kidney releases EPO. EPO controls the expression of hepcidin in the body through 2 aspects: 1. EPO stimulates iron to be incorporated into the erythrocyte precursors in the bone marrow and reduces the iron saturation of transferrin, thus inhibiting the expression of hepcidin by reducing iron load. 2. EPO plays an additional role in reducing hepcidin by stimulating bone marrow erythroblasts to produce ERFE, which partially inhibits the BMP-SMAD pathway in the liver. In addition, hypoxia-inducible factors can directly inhibit the transcription of hepcidin, thus interfering with BMP signal transduction and inhibiting hepcidin activity (Fig. 2).

## 3. The role of hepcidin

The transport of iron to every cell in the body depends on binding to plasma transferrin. Iron is supplied in circulation by macrophages, duodenal cells, and hepatocytes, which release iron into circulation through FPN.^[23]^ Hepcidin inhibits iron entry into circulation by regulating FPN on duodenal cells, macrophages, placental syncytiotrophoblast cells, and hepatocytes to induce their degradation. FPN is the main exporter of extracellular iron, and its effect reduces the outflow of iron from cells and significantly affects the content of iron in the liver and circulating iron. The synthesis of FPN is regulated by cellular hypoxia, iron concentration, heme concentration, and the inflammatory signaling pathway.^[24]^ The process is also regulated by hypoxia-inducible factor 2α and heme-dependent transcription factors and by the IRE/IRP regulatory network and miR-485-3p after transcription. At the molecular level, the binding of hepcidin to FPN1 leads to ubiquitination and internalization of the receptor and lysosome degradation.^[25,26]^

In addition to directly affecting FPN, hepcidin may also control iron output from FPN1 through gated transporters without inducing endocytosis. Regardless of what causes the decrease in hepcidin, it will cause an increase in iron in the blood. If the buffering capacity of serum Tf is the saturated, nonTf binding of iron occurs, and labile plasma iron is formed.^[27]^ Labile plasma iron is eventually transferred to the parenchyma cells of the liver, resulting in iron overload.

## 4. Iron overload and liver disease

### 4.1. Chronic hepatitis B (CHB)

Chronic hepatitis B virus (HBV) infection often develops into liver cirrhosis, both of which are the main risk factors for hepatocellular carcinoma (HCC). Iron overload is a common symptom in patients with CHB, with up to 27.1% of patients having elevated Tf saturation and 48.7% having liver iron deposition.^[28]^ Furthermore, the concentration of serum ferritin decreases after treatment with antiHBV drugs.^[29]^ The level of serum iron in patients with positive serum HBsAg is higher than that in patients without HBsAg, regardless of the level of transaminase.^[30]^ In patients with CHB, iron storage involves elevated serum iron and ferritin levels and continuous accumulation of iron in the liver. Iron can also affect the proliferation of HBV. Many studies have explored the relationship between iron overload and the life cycle of HBV, and different results have been obtained. Some people have found that iron promotes the life cycle of HBV,^[31,32]^ while others have found that iron limits the life cycle of HBV.^[33]^ In 1983, a study of 44 HBV patients^[34]^ found that higher levels of serum ferritin before HBV infection increased the likelihood of persistent infection, suggesting that iron may promote HBV infection.

Moreover, G. Sebastiani^[35]^ investigated liver iron deposition and the serum iron index in 205 consecutive patients with hepatitis B and hepatitis B complicated with hepatitis D (from hepatitis D virus [HDV] infection). Compared with the patients with only HBV infection, the patients with combined HDV and HBV infection had higher levels of GGT, more severe fibrosis, and more severe iron deposition (*P* < .0001). Liver iron deposition and an elevated serum iron index are common in well-compensated CHB infections, especially in males and patients with HDV. Since the progression of HBV/HDV-related liver disease is usually faster than that caused by HBV infection alone, iron overload may be one of the factors leading to its severity.

The mechanism of iron overload in CHB patients may be related to hepcidin,^[36]^ liver injury,^[30]^ viral activity,^[37]^ miR-122,^[38]^ ROS,^[39]^ IL-6, and/or other inflammatory factors. Among them, ROS are most closely related to iron overload because ROS can play a role in the signal transduction cascade by activating transcription factors, including STAT-3. The STAT-3 pathway is responsible for activating hepcidin transcription in hepatocytes.^[36]^ In addition, IL-6 is a strong stimulator of hepcidin expression. Many studies have shown that the level of IL-6 in patients with CHB is higher than that in healthy controls.^[36]^ IL-6 can effectively enhance the expression of hepcidin through the STAT-3 pathway from the hepatocyte membrane activated by the IL-6 receptor. In addition, viral or bacterial infection can stimulate the synthesis of hepcidin, and the increase in hepcidin may be attributed to HBV activity.^[40]^ According to the above studies, hepcidin is likely not the main cause of iron overload in patients with CHB, but a change in hepcidin level is related to the progression of HBV infection.^[41]^

### 4.2. Chronic hepatitis C (CHC)

As with CHB, up to 40% of patients with CHC are diagnosed with elevated total iron reserves.^[42–44]^ Available data show that HBV and hepatitis C virus (HCV) have different effects on hepcidin. HBV increases the expression of hepcidin, while HCV decreases its expression.^[44]^ Furthermore, some studies have shown that chronic viral hepatitis inhibits the synthesis of hepcidin by inducing oxidative DNA damage.^[45,46]^ Compared with that caused by HCV, the oxidative damage caused by HBV is relatively mild.^[47]^ Iron deposition was found in hepatocytes and reticular endothelial cells of patients with CHC.^[48]^ Similar to other viruses, HCV needs host cell components to proliferate, and iron is one of the most critical components. Many studies have explored the relationship between iron overload and the life cycle of HCV, and different results have been obtained. Some studies have found that iron promotes HCV replication,^[49,50]^ while others have shown that iron inhibits it.^[51]^

**Figure 3.**
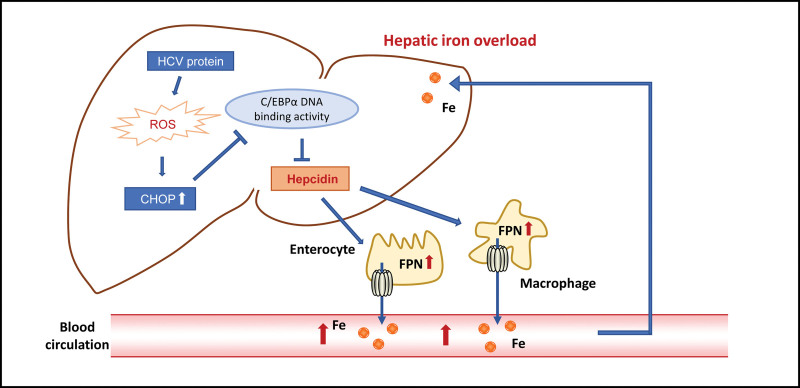
HCV reduces hepcidin expression resulting in hepatic iron overload. HCV. HCV protein can induce ROS to decrease the transcription of hepcidin by inducing C/EBPα DNA binding activity. Then excess iron enters the blood circulation through the FPN of enterocyte and macrophage, which eventually leads to hepatic iron overload. C/EBPα = CCAAT enhancer-binding protein α, FPN = ferroportin, ROS = reactive oxygen species.

HCV infection is closely related to abnormal glucose.^[52]^ The key factor affecting glucose in this disease is abnormal iron metabolism. The third National Health and Nutrition Examination Survey shows that people over the age of 40 years who are infected with HCV are more than 3 times more likely to develop type 2 diabetes than those who are not infected with HCV.^[53]^ In addition, Metha et al^[54]^ reported that HCV infection might increase the risk of developing different forms of diabetes, such as type 2 diabetes, in patients with family history and impaired glucose tolerance. Shintani et al,^[55]^ using HCV core transgenic mice, showed direct experimental evidence of the contribution of HCV to the development of insulin resistance. Therefore, there is increasing evidence to support the concept that HCV infection is a risk factor for type 2 diabetes, but the specific mechanism by which HCV causes type 2 diabetes is not fully understood.^[56]^ It is speculated that steatosis, proinflammatory cytokines, and iron overload may be closely related to the glucose abnormalities associated with HCV infection. The mechanism of hepatic iron overload in patients with chronic HCV infection is unclear. However, recent studies have shown that HCV improperly suppresses the expression of hepcidin, inducing ROS to increase iron levels in mouse liver by lowering hepcidin transcription^[57]^ and increasing histone deacetylase activity to increase hepcidin downregulation (Fig. 3).

Studies on HCV-infected and uninfected chimpanzees have shown that iron overload does aggravate liver damage, and HCV infection increases the susceptibility of the liver to damage after iron overload. An increased liver iron deposition is associated with more advanced liver fibrosis in patients with chronic hepatitis.^[58]^ Recently, it has been prospectively shown in a cohort of patients treated with long-term antiviral therapy for cirrhosis that iron staining in hepatocytes and portal vein cells can be assessed to predict progression and outcomes in advanced chronic hepatitis patients (patients with Child–Pugh score > 7, ascites, encephalopathy, varicose bleeding, spontaneous bacterial peritonitis, HCC, and death). Therefore, iron is a cofactor affecting the severity and progression of chronic hepatitis. The association between a significant increase in liver iron accumulation in hereditary hemochromatosis and the occurrence of liver cancer has been studied in detail. It has been confirmed that there is a correlation between the 2. However, whether the mild to moderate increase in liver iron accumulation contributes to the development of HCC in patients with HCV-related chronic liver disease remains to be clarified.

### 4.3. Alcoholic liver disease (ALD)

ALD refers to liver damage caused by excessive drinking, ranging from benign steatosis to steatohepatitis and cirrhosis.^[59]^ Compared with iron overload in HBV and HCV infection, iron overload is found to occur in many alcoholic liver diseases, especially alcoholic hepatitis complicated with liver cirrhosis.^[60]^ It is well known that the concentration of serum ferritin is proportional to the concentration of iron in the liver,^[61]^ and drinking alcohol can significantly increase the concentration of serum ferritin.^[62]^ Compared with other chronic liver diseases, such as chronic viral hepatitis, ALD with alcohol abuse is more likely to increase the level of serum ferritin. In contrast, abstinence from alcohol leads to a rapid decrease in it.^[62]^

Iron has been identified as an independent risk factor for survival and liver cancer in patients with ALD.^[63]^ Many researchers have tried to understand the relationship between ALD and iron overload. Among them, Harrison-Findik et al^[64]^ constructed a mouse model and found that alcohol can inhibit the activation of C/EBP α and thus inhibit the expression of hepcidin mRNA. Iron can upregulate hepcidin transcription during iron overload, but alcohol can block this process. Costa-Matos et al^[65]^ compared and analyzed the indexes of 45 patients with ALD and healthy controls and concluded that the ratio of hepcidin mRNA expression to serum ferritin concentration in ALD patients was decreased significantly. In addition, the downregulation of hepcidin is caused by alcohol metabolism,^[66,67]^ not by ethanol itself,^[67]^ because the inhibition of alcohol metabolic enzymes by 4-methylpyrazole eliminates the effect of alcohol on hepcidin.^[65]^ In addition, Dostalikova-Cimburova et al^[68]^ studied the expression of genes encoding iron transport molecules in the duodenum and hepcidin in patients with ALD. They found that disordered iron metabolism in patients with ALD was most likely due to the downregulation of hepcidin expression, which led to the upregulation of iron transporter expression in the duodenum.

In addition, similar to the case in chronic viral hepatitis, a variety of factors involved in the mechanism by which alcohol affects the expression of hepcidin have been proposed, among which ROS,^[69]^ IL-6,^[67]^ transferrin 1 and FPN^[66]^ are supported by evidence in mouse experiments.

Recently, Nahon et al^[70]^ evaluated the effect of serum hepcidin on the long-term survival of patients with alcoholic cirrhosis by analyzing the prognosis of 237 patients with alcoholic cirrhosis. It was found that patients with low concentrations of hepcidin had a higher risk of developing liver cancer, and the decrease in hepcidin levels was independently associated with death.

### 4.4. Nonalcoholic fatty liver disease (NAFLD)

NAFLD and its more severe form, nonalcoholic steatohepatitis (NASH), are serious global health problems.^[71]^ The estimated prevalence of NAFLD worldwide is 20% to 50%,^[72]^ and the pathogenesis of NAFLD is considered to be multifactorial. Approximately 30% of patients with NAFLD accumulate too much iron,^[73]^ which is called dysmetabolic-hepatic iron overload syndrome.^[74]^ Up to 60% of NASH patients have increased serum ferritin levels, but there are lesser increases in liver iron content, and there is no correlation between serum ferritin and liver iron concentration in NASH.^[75]^

In NAFLD, many factors lead to disordered iron metabolism, and an increase in serum ferritin concentration is an independent predictor of advanced liver fibrosis in patients with NAFLD.^[76]^ An unhealthy diet is one of the main risk factors for the development and progression of NAFLD. The increase in iron concentration in hepatocytes caused by diet and inflammatory stimulation promotes the production of hepcidin in the liver of patients with NAFLD. In addition, glucose can stimulate pancreatic β-cells to release hepcidin.^[77]^ Therefore, serum hepcidin in patients with NAFLD is higher than that in normal people, and the increase in serum hepcidin leads to a decrease in FPN expression and cell iron output,^[78]^ which further stimulates iron deposition in hepatocytes. In addition to the secondary effect of hepcidin on iron overload, the main cause of iron overload in patients with NAFLD is a diet rich in free fatty acids and monosaccharides. free fatty acids can indirectly or directly affect the absorption and release of iron through ferritin.^[79,80]^

In general, these factors enhance the accumulation of iron in cells and accelerate the process of fibrosis. Excessive iron and lipids (resulting from the activation of inflammatory cascades caused by lipid peroxidation^[81]^) may exacerbate fibrosis because they can lead to significant oxidative damage. Therefore, compared with nonNASH patients, NASH patients with iron overload show a higher fibrosis grade and higher liver function test results.^[81]^ Therefore, iron plays a pathogenic role in NAFLD and is one of the many factors that determine the progression from NASH to fibrosis.^[79]^

Sorrentino et al,^[81]^ through a retrospective analysis of 153 patients with NASH-related cirrhosis, found that liver iron deposition was more frequent and more serious in patients with liver cancer than in those without liver cancer. Liver iron overload may be related to the occurrence of HCC in patients with NASH-related cirrhosis. Recently, Corradini et al^[82]^ used next-generation sequencing technology to explore genetic variations related to iron metabolism, especially variations related to altered plasma ceruloplasmin and hyperferremia, and increased liver iron storage in patients with NAFLD and to explore the genetic susceptibility to liver disease.

### 4.5. Hepatocellular carcinoma

As a common lethal malignant tumor, HCC has attracted worldwide attention in recent years. Sikorska et al^[83]^ found that an increase in iron reserves in the liver may lead to the progression of liver injury and fibrosis and is associated with a high risk of the development of HCC. Liver cirrhosis is a major risk factor for HCC, as cirrhosis is present in 70% of HCC cases worldwide. In patients with liver cirrhosis without a clear background, iron deposition in liver regenerative nodules is associated with HCC.^[84]^ Excessive liver iron can lead to tumor transformation in cirrhotic livers or lead to liver cancer. In addition, the prevalences of iron overload and liver cancer are higher in patients with viral hepatitis C or hepatitis B cirrhosis.^[85]^ An increase in serum transferrin saturation is associated with increased incidences of liver cirrhosis and liver cancer, especially in patients with high alcohol intake.^[86]^ Yanyan Wei et al compared the hematological parameters, HBV DNA, and liver biochemical indexes of CHB patients, HBV-related liver cirrhosis patients, and HBV-related HCC patients with those of healthy individuals. The Child–Pugh grade and BCLC stage of the patients with HBV-related HCC were determined. The results showed that the level of serum iron was negatively correlated with the size of the tumor. However, the serum iron levels of most patients with HBV-related HCC are still within the normal range. Therefore, the functional iron in peripheral blood may be low or normal, but the iron content in liver tissue may be higher.^[87]^ In addition, it has been reported that sorafenib combined with the iron chelator deferasirox can enhance the apoptosis of HCC cells induced by sorafenib.^[88]^ Therefore, iron chelation may become a new adjuvant therapy for HCC.

The serum iron level is an independent risk factor for the survival of HBV-related HCC patients. In contrast, a lower AFP level, smaller tumor size, and better BCLC stage are independent protective factors for the survival of HBV-related HCC patients.^[87]^

In addition to liver cirrhosis, hereditary hemochromatosis (HH) is another disease caused by the liver iron overload that is prone to developing into liver cancer. Approximately 8% to 10% of patients with HH develop HCC. The genetic susceptibility of HH patients to dietary iron overabsorption is almost always caused by homozygous C282Y mutation of the HFE gene, which leads to low secretion of hepcidin and iron overload.

The expression of hepcidin mRNA is generally inhibited in HCC, which is obvious in cancerous tissues but not in noncancerous tissues and has nothing to do with the degree of tumor differentiation, the tumor stage, or tumor recurrence.^[89]^ The HAMP promoter that controls the expression of the HAMP gene, which encodes hepcidin, contains binding motifs for C/EBP,^[90]^ hepatocyte nuclear factor 4,^[90]^ STAT-3,^[91]^ and SMAD4.^[92]^ Weizer-Stern et al^[93]^ have shown that the promoter region of the HAMP gene contains a presumed p53 response element, and its expression is caused by the activation of p53. Therefore, activation of p53 stimulates the expression of hepcidin. The decrease in hepcidin in patients with HCC may be related to a decrease in p53 activity related to the development of HCC.

It has been reported that most HCC patients with noncirrhotic liver (NCL) have the histological and biochemical iron overload in nontumor liver tissues.^[94]^ Jean-FrkdCric Blancl^[95]^ looked for the relationship between iron metabolism and HCC in HCC patients in NCL and retrospectively studied 35 HCC patients in NCL. In this small series of studies, 50% of HCC patients with NCL had liver iron overload at the histological level. Based on the above research results, there is a clear relationship between HCC and iron overload, and further research on the mechanism is needed.

### 4.6. Iron reduction therapy

Iron overload caused by various factors causes the occurrence of many diseases mentioned above, so exploring treatment methods for such diseases according to the above mechanism has become a major focus of research.

Interferon (IFN): IFN activates a variety of immune systems in the body and is used in patients with diseases such as hepatitis B, hepatitis C, and malignant tumors. Kazuhiko et al^[96]^ administered mouse IFNα to mice and then collected spleen, bone marrow, and liver samples. According to real-time reverse transcription-polymerase chain reaction and Western blotting analyses, the mRNA and protein expression levels of iron metabolism-related genes were analyzed. Gene expression analysis indicated that the expression of hepcidin in the liver was highly upregulated after IFNα treatment. In vitro analysis of IFNα-treated primary hepatocytes and human hepatoma-derived cells showed that hepcidin mRNA was upregulated, as was STAT-3. These findings prove that IFN is involved in the upregulation of hepcidin. Harald^[97]^ assessed hepatic iron concentration and serum iron parameters to determine the response of CHC patients to standard IFN and pegylated IFN/ribavirin therapy. The results showed that baseline serum ferritin levels were an independent predictor of successful virus elimination in patients with CHC who received standard IFN or pegylated IFN/ribavirin therapy.

Phlebotomy: Phlebotomy has been used since the early stage of medicine in iron overload diseases. With the development of modern medicine, this treatment has become less common, but it is still important.^[98]^ The principle of phlebotomy is to reduce the number of human red blood cells to stimulate the bone marrow to absorb iron for hematopoiesis to compensate for the lack of red blood cells in the body. Phlebotomy can effectively reduce disease progression and prolong the survival time of patients with iron overload symptoms.^[99]^ The American society of hematology recommends that phlebotomy is started when ferritin levels are higher than 300 μg/L in men or 200 μg/L in women with fertility potential. The American association for the study of liver diseases and the European association for the study of the liver recommend that treatment be started as soon as ferritin levels are above the upper limit of normal.^[100]^ In the absence of randomized controlled trials, these recommendations are based on clinical evidence that iron removal before the onset of liver cirrhosis and diabetes is associated with reduced morbidity and mortality. Some features, including liver fibrosis, may be ameliorated by phlebotomy.^[101]^ Nirei et al^[102]^ reported that phlebotomy could reduce the incidence of HCC in patients with HCV infection.

Chelating agents: For many years, phlebotomy was the only strategy for removing excess iron. However, because this method has many disadvantages, chelation therapy has emerged as another way to reduce toxic iron levels. Deferasirox is a novel iron removal drug that has been proven to eliminate iron overload in patients with HFE-related hemochromatosis effectively, but it has some side effects on the liver and kidney.^[103]^ Studies have shown that the deferasirox combination can enhance the efficacy of sorafenib in the treatment of liver cancer.^[88]^ In the future, strategies employing iron-chelating agents combined with iron-related targeted drugs may become a new trend in treatment.

Erythrocytapheresis: Erythrocytapheresis is a technique that selectively removes red blood cells and returns blood components such as white blood cells, platelets, and plasma. This selective removal of red blood cells can increase iron removal and reduce hemodynamic events associated with blood collection.^[104]^

## 5. Conclusions

The study of hepcidin, the main regulator of iron homeostasis in the body, has greatly improved our understanding of iron biology. It is clear that hepcidin plays an important role in absorbing, storing, and releasing iron. The concentration of hepcidin is strictly controlled, and hepcidin dysregulation is the basis of many human diseases. The regulation of hepcidin is multifaceted and complex, and many positive and negative regulatory factors converge to fine-tune its expression. Clear regulatory pathways of hepcidin include the BMP-SMAD pathway, IL-6 pathway, and EPO-ERFE axis. As the main producer of hepcidin and iron-related hormones and the main iron pool in the body, the liver plays a central role in the regulation of human iron homeostasis. However, when the buffered iron storage and antioxidant capacities of organs are overcome, many liver diseases related to iron-driven toxicity may occur. Given the involvement of iron in the risk of liver failure, hepatitis, and HCC, the role of iron in advanced liver disease needs to be further studied. A better understanding of the mechanisms leading to iron homeostasis disorder and the clinical consequences is necessary for the treatment of iron overload and the improvement of prognosis in patients with liver cirrhosis.

## Author contributions

**Conceptualization:** Yong-Chang Zheng.

**Data curation:** Kaige Deng, Jiaxin Wei, Zhenting Liu.

**Writing – original draft:** Haoran Zheng, Fan Yang.

**Writing – review & editing:** Yong-Chang Zheng, Haifeng Xu.
